# Blood platelets stimulate cancer extravasation through TGFβ-mediated downregulation of PRH/HHEX

**DOI:** 10.1038/s41389-020-0189-0

**Published:** 2020-02-04

**Authors:** Eudmar Marcolino, Yusra Hasan Siddiqui, Marion van den Bosch, Alastair W. Poole, Padma-Sheela Jayaraman, Kevin Gaston

**Affiliations:** 1 Division of Cancer and Stem Cells, School of Medicine, University of Nottingham, Nottingham, NG2 7UH UK; 2 School of Biochemistry, University of Bristol, Bristol, BS8 1TD UK; 3 School of Physiology, Pharmacology and Neuroscience, University of Bristol, Bristol, BS8 1TD UK; 4 Division of Immunity and Infection, School of Medicine, University of Birmingham, Birmingham, B15 2TT UK

**Keywords:** Cancer microenvironment, Extracellular matrix

## Abstract

Cancer cells go through a process known as epithelial–mesenchymal transition (EMT) during which they acquire the ability to migrate and invade extracellular matrix. Some cells also acquire the ability to move across a layer of endothelial cells to enter and exit the bloodstream; intra- and extravasation, respectively. The transcription factor PRH/HHEX (proline-rich homeodomain/haematopoietically expressed homeobox) controls cell proliferation and cell migration/invasion in a range of cell types. Our previous work showed that PRH activity is downregulated in prostate cancer cells owing to increased inhibitory PRH phosphorylation and that this increases cell proliferation and invasion. PRH inhibits migration and invasion by prostate and breast epithelial cells in part by activating the transcription of Endoglin, a transforming growth factor β (TGFβ) co-receptor. Here we show that depletion of PRH in immortalised prostate epithelial cells results in increased extravasation in vitro. We show that blood platelets stimulate extravasation of cells with depleted PRH and that inhibition of TGFβ signalling blocks the effects of platelets on these cells. Moreover, TGFβ induces changes characteristic of EMT including decreased E-Cadherin expression and increased Snail expression. We show that in prostate cells PRH regulates multiple genes involved in EMT and TGFβ signalling. However, both platelets and TGFβ increase PRH phosphorylation. In addition, TGFβ increases binding of its effector pSMAD3 to the PRH/HHEX promoter and downregulates PRH protein and mRNA levels. Thus, TGFβ signalling downregulates PRH activity by multiple mechanisms and induces an EMT that facilitates extravasation and sensitises cells to TGFβ.

## Introduction

During metastasis, cancer cells undergo a change in phenotype known as epithelial–mesenchymal transition (EMT). In this process, the cancer cells lose normal cell–cell interactions and apico-basal polarity and they become migratory and invasive (reviewed by Shibue and Weinberg 2017^1^). This enables cancer cells to migrate away from their site of origin, and it facilitates intravasation, passage of cells across the endothelial cell barrier and into a blood vessel and extravasation, passage from a blood vessel into tissues. Once in their new location, the reverse mesenchymal–epithelial transition is thought to take place enabling the formation of secondary tumours. EMT in cancer cells can also alter the behaviour of surrounding cells that have not undergone EMT and alter the response of cancer cells to chemotherapy^2,3^. Multiple signalling molecules are known or suspected to be important in the induction of EMT in pre-cancer cells in the tumour microenvironment or in the maintenance of the mesenchymal state in blood vessels, including factors released by immune cells^4,5^.

Inflammation has a role in prostate tumourigenesis through several mechanisms, including the creation of a tissue microenvironment capable of inducing cell replication, and the induction of neo-angiogenesis^6^. Multiple cytokines have been identified as potential mediators between prostatic inflammation and prostate cancer risk and aggressiveness including IL-6, IL-2 and transforming growth factor β (TGFβ)^7,8^. Although TGFβ has both anti- and pro-proliferative roles; the current consensus is that as tumours become aggressive, TGFβ signalling responses are altered, resulting in the promotion of metastasis. Indeed, treatment of multiple cell lines with TGFβ is sufficient to induce EMT^9,10^. Once they enter blood vessels, cancer cells can bring about the aggregation of platelets, triggering their activation and the release of numerous small molecules and proteins, including TGFβ, adenosine triphosphate (ATP), vascular endothelial growth factor (VEGF) and platelet-derived growth factor (PDGF)^11,12^. Platelets also protect cancer cells from immune responses and shear stress, and they can alter cancer cell behaviour, enabling extravasation and other events important in metastasis^13^. Moreover, platelets are also present in the tumour microenvironment as a result of their extravasation^14,15^. Platelet-released signalling molecules could therefore also induce or exacerbate EMT in cancer cells as well as in pre-cancerous cells, and this may also promote extravasation and metastasis.

The transcription factor PRH/HHEX (proline-rich homeodomain/haematopoietically expressed homeobox) is essential for the formation of many organs during embryonic development^16,17^. PRH is expressed in many tissues in the adult and downregulation of PRH protein expression and/or aberrant subcellular localisation of PRH is associated with breast, prostate, thyroid, and liver tumours^18^. PRH inactivation is common to a number of cancer types but the mechanisms of PRH inactivation vary in different disease states. PRH mRNA levels are reduced in breast cancer cells and in poorly differentiated hepatocellular carcinomas^19,20^. In addition, PRH protein is present in the nucleus and cytoplasm in normal breast epithelial cells and normal thyroid tissues but in breast and thyroid cancer cells, the protein is found predominantly in the cytoplasm^20–22^. Our recent studies have shown that in prostate and breast cancer cells PRH is inactivated by protein kinase CK2-dependent phosphorylation of the PRH homeodomain^23,24^. Phosphorylation by CK2 prevents PRH from binding to DNA and alters nuclear retention. In addition, phosphorylation by CK2 targets PRH for processing by the proteasome creating a truncated PRH phosphoprotein that is capable of acting as a transdominant inhibitor of full-length transcriptionally active PRH^24^.

Depletion of PRH in immortalised but otherwise normal prostate PNT2-C2 epithelial cells and normal immortalised breast MCF10A cells results in increased cell migration and cell invasion^25^. In contrast, the overexpression of PRH in prostate cancer PC3 cells and breast cancer MCF-7 cells inhibits cell proliferation and cell migration/invasion. Furthermore, PRH overexpression inhibits extravasation by PC3 cells in an in vitro assay in which cancer cells traverse a layer of confluent endothelial cells grown on Matrigel^25^. In addition, we showed recently that PRH is inactivated in benign prostatic hyperplasia (BPH) and prostate cancer cells via increased protein phosphorylation^26^. This suggests that inactivation of PRH could be important in the acquisition of a mesenchymal phenotype during prostate tumourigenesis. Here, we show that PNT2-C2 epithelial cells with depleted PRH are able to traverse a layer of endothelial cells in an in vitro extravasation assay. Importantly, the PRH-depleted cells are more responsive to the stimulatory effects of blood platelets in this assay than control cells. We show that TGFβ released from platelets is responsible in part at least for the stimulation of in vitro extravasation. Moreover, TGFβ induces EMT-like changes in PNT2-C2 cells and prostate cancer cells and this is accompanied by increased PRH phosphorylation and decreased PRH protein and mRNA levels. These data indicate that TGFβ signalling inactivates PRH using multiple mechanisms resulting in increased the sensitivity to TGFβ signalling.

## Results

### PRH depletion increases in vitro extravasation

**Fig. 1:**
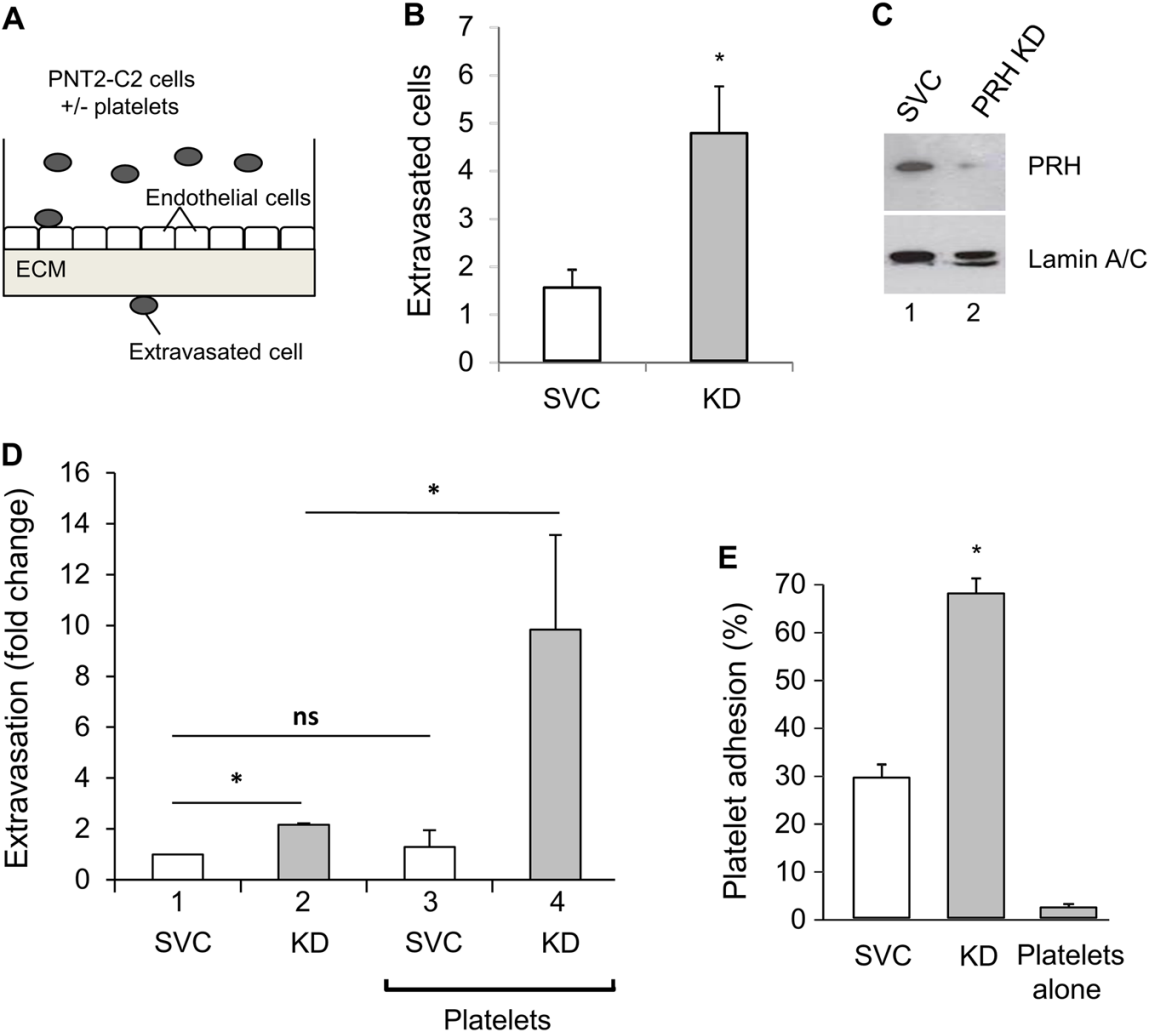
Blood platelets stimulate in vitro extravasation by immortalised prostate epithelial cells. **a** A schematic representation of an in vitro extravasation assay. **b** PNT2-C2 cells expressing PRH shRNA (KD) or a scrambled vector control shRNA (SVC) were placed in a transwell chamber containing a confluent layer of HuVECs growing on Matrigel. After 48 h, the number of extravasated cells was determined by counting cells on the lower side of the transwell filter using microscopy. Cells per field, *n* = 3 independent experiments, mean and standard deviation (M + SD), *p < 0.01. **c** Western blot showing PRH protein levels in the cells from **b** with Lamin A/C as loading control. **d** The experiment shown in **b** was repeated with SVC and PNT2-C2 PRH KD cells that had been pre-incubated with blood platelets (1:1 cells: platelets) for 24 h. M + SD from three independent experiments, *p < 0.01, ns = not significant. **e** Platelet adhesion to control PNT2-C2 cells and PRH KD cells was measured using Calcein-labelled platelets. Percentage platelet adhesion = (remaining fluorescence−blank)/(total fluorescence−blank) × 100. M + SD, *n* = 3, *p < 0.01.

To determine whether depletion of PRH in prostate epithelial cells enables extravasation, we made use of an in vitro assay in which human umbilical vein endothelial cells (HuVECs) are plated onto a layer of Matrigel in transwell chambers and allowed to form a barrier (Fig.1a). Each layer of HuVECs was tested to ensure that it was impermeable to fluorescein isothiocyanate (FITC)-labelled dextran before use. Normal immortalised prostate epithelial PNT2-C2 cells transfected with plasmids expressing either a scrambled vector control (SVC) shRNA or PRH shRNA and grown for 10 days in puromycin selection were then plated onto the confluent HuVECs. After 48 h, the number of extravasated cell on the lower side of the transwell filter was determined using microscopy. Figure 1b shows that PRH knockdown (KD) using shRNA resulted in a significant increase in extravasation in this assay. PRH levels in the control and PRH KD cells are shown in Fig. 1c and demonstrate effective KD.

### Platelets stimulate in vitro extravasation

Blood platelets are known to stimulate extravasation of cancer cells^27,28^. To examine the effects of platelets on extravasation by PNT2-C2 cells and PNT2-C2 with knocked down PRH, we pre-incubated cells with platelets (1:1 ratio) for 24 h and then measured extravasation over a further 24 h. Pre-incubation with platelets had little or no effect on the ability of control cells to traverse the endothelial layer (Fig.1d). However, pre-incubation with platelets resulted in a fivefold increase in extravasation by the PRH KD cells (Fig.1d). In these assays, the permeability of the endothelial cell layer to FITC-labelled dextran was not increased by exposure to platelets, suggesting that the platelets act directly on the PRH KD cells. In keeping with this conclusion the adherence of labelled platelets to PNT2-C2 cells is increased following PRH KD (Fig.1e). Taken together, these data suggest that endogenous PRH is likely to repress invasion and extravasation of normal cells and that loss of PRH activity in BPH and in prostate cancer cells contributes to the acquisition of these abilities. Experiments in breast cancer cells support this view and indicate that this consequence of PRH inactivation is not confined to prostate cells: MCF-7 breast cancer cells with knocked down PRH display increased extravasation (Fig. S1). Moreover, pre-incubation with platelets results in a significant increase in extravasation (Fig. S1).

### ATP and TGFβ signalling promote extravasation

**Fig. 2:**
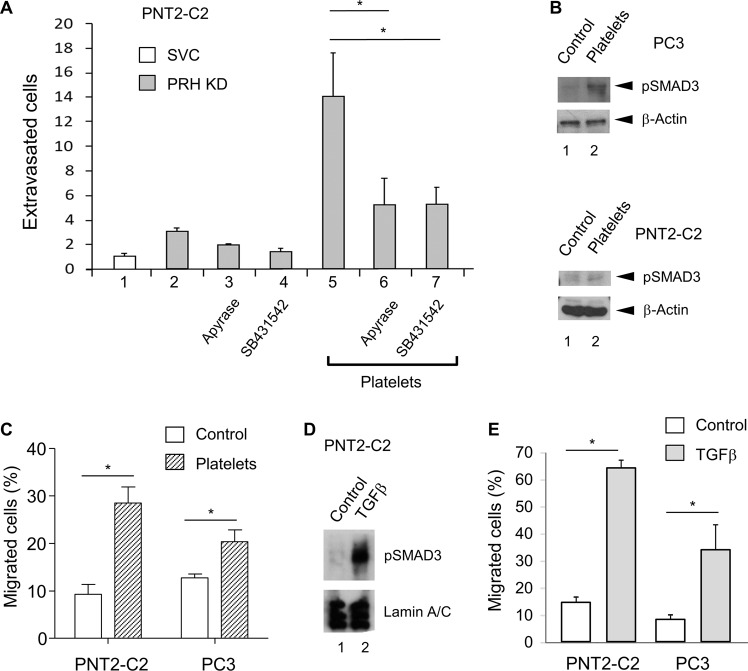
ATP and TGFβ signalling increase prostate cell extravasation. **a** PNT2-C2 cells expressing PRH shRNA (KD) or a scrambled vector control shRNA (SVC) were placed in a transwell chamber containing a confluent layer of HuVECs growing on Matrigel. Cells were pre-treated with platelets as in Fig. 1d in the absence and presence of apyrase treatment (columns 5 and 6) and in the absence and presence of 3 mm SB431542 (columns 5 and 7). After 24 h the number of extravasated cells was determined as described in Fig. 1b. Cells per field, *n* = 3 independent experiments, M + SD, *p < 0.01. **b** Immortalised prostate PNT2-C2 cells and prostate cancer PC3 cells were incubated with platelets (1:1) for 24 h. pSMAD3 levels were then determined using western blotting with β-Actin as loading control. **c** Cell migration over 18 h was then examined using a transwell chemotaxis assay. The percentage of cells migrated was determined by counting the cells on the top and bottom surfaces of the transwell filter using microscopy. Three independent experiments, M + SD, *p < 0.01. **d** PNT2-C2 cells were treated with 5 ng/ml TGFβ or vehicle for 48 h. Western blotting was then used to measure pSMAD3 levels using Lamin A/C as loading control. **e** PNT2-C2 cells and prostate cancer PC3 cells were treated with 5 ng/ml TGFβ for 48 h as above. Cell migration was then assayed as above. Three independent experiments, M + SD, *p < 0.01.

Platelets release multiple growth factors and other signalling molecules that could stimulate extravasation by PRH-depleted PNT2-C2 cells and MCF-7 cells^29,30^. We first tested whether ATP release by platelets is important in the response of PNT2-C2 PRH KD cells. Treatment with apyrase, an ATP diphosphohydrolase, reduced the stimulatory effect of platelets on the PRH KD cells by > 50% (Fig. 2a), indicating that ATP signalling increases extravasation by these cells. As our previous work has shown that PRH directly regulates transcription of the gene encoding the TGFβ co-receptor Endoglin^25^, we next tested whether TGFβ signalling is important in this context. We incubated PNT2-C2 PRH KD cells with platelets in the presence and absence of SB431542, an inhibitor of TGFβ signalling via the activin type I receptors ALK5/4/7^31^. Treatment with this inhibitor also reduced the stimulatory effect of platelets on the PRH KD cells by > 50% (Fig. 2a). To confirm that platelets activate the TGFβ signalling pathway in these cells and in prostate cancer PC3 cells, we treated the cells with platelets and examined the levels of pSMAD3, an effector of TGFβ signalling. In both cell types, platelet treatment brings about an increase in the levels of pSMAD3 (Fig. 2b). As expected, treatment with platelets also increased cell migration in both PNT2-C2 cells and PC3 cells (Fig. 2c). We next treated PNT2-C2 cells with TGFβ and examined pSMAD3 levels and cell migration. Treatment of these cells with TGFβ increased pSMAD3 levels (Fig. 2d) and increased cell migration by around fourfold (Fig. 2e). We conclude that TGFβ and ATP released from platelets are responsible, in part, at least for the effects of platelets on PNT2-C2 cell migration and extravasation. As incubation with platelets increased the migration of prostate cancer PC3 cells, we also examined the effects of TGFβ on these cells. As expected, treatment with TGFβ also increased the migration of PC3 cells (Fig. 2e).

### TGFβ induces EMT-like changes in PNT2-C2 cells

**Fig. 3:**
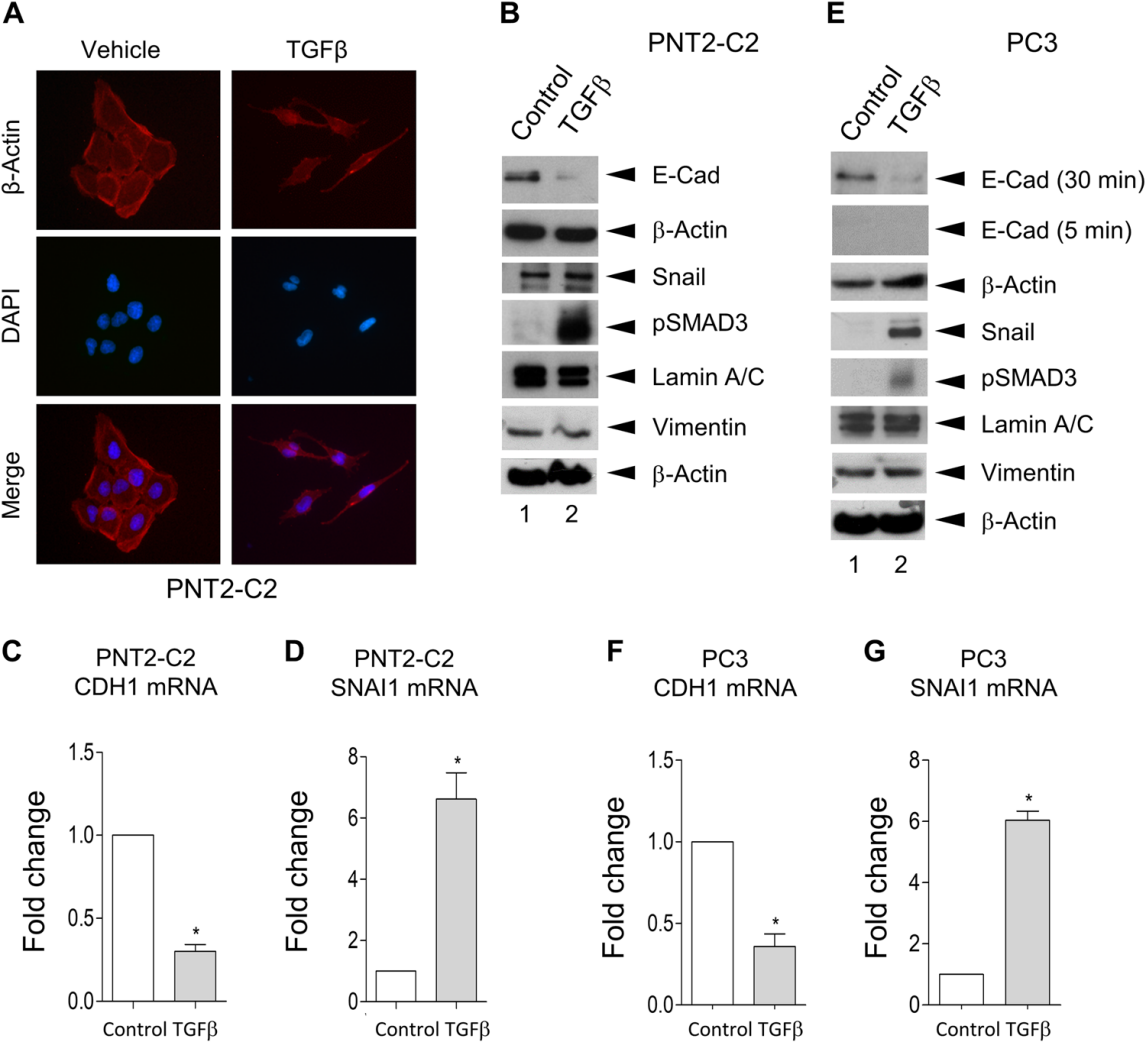
TFGβ induces EMT-like changes in PNT2-C2 cells and PC3 cells. **a**–**d** Immortalised prostate PNT2-C2 cells were treated with 5 ng/ml TGFβ for 48 h. Cell morphology was then assessed using immunofluorescence to stain for β-Actin and DAPI to stain for DNA **a**. EMT markers (E-Cadherin, Snail and Vimentin) and pSMAD3 levels were examined using western blotting with β-Actin and Lamin A/C as loading controls **b**. Following mRNA extraction qRT-PCR was used to determine CDH1 and SNAI1 mRNA levels **c** and **d**, respectively. Three independent experiments with PCR reactions performed in triplicate, M + SD, *p < 0.01. **e**–**g** Prostate cancer PC3 cells were treated with 5 ng/ml TGFβ for 48 h as above. EMT markers and pSMAD3 levels were examined using western blotting as above **e**. qRT-PCR was used to determine CDH1 and SNAI1 mRNA levels as above (**f** and **g**, respectively). *p < 0.01.

To examine the effects of TGFβ on PNT2-C2 cells in more detail, we next examined cell morphology and the expression of markers of EMT. We examined the morphology of untreated and TGFβ-treated cells using immunofluorescence with antibodies that stain β-Actin and 4′,6-diamidino-2-phenylindole (DAPI) to stain DNA. Untreated PNT2-C2 cells are typically rounded and show extensive cell–cell contacts characteristic of epithelial cells (Fig. 3a). In contrast, TGFβ-treated PNT2-2C cells display an elongated, spindle-shaped morphology typical of mesenchymal cells (Fig. 3a). Western blotting reveals that expression of the epithelial marker and cell adhesion protein E-Cadherin is substantially reduced in the treated cells (Fig. 3b) and quantitative PCR reveals that the CDH1 mRNA encoding E-Cadherin is significantly reduced in these cells (Fig. 3c). Expression of the mesenchymal marker protein Snail is somewhat elevated in the TGFβ treated cells (Fig. 3b). However, the SNAI1 mRNA encoding this protein is upregulated by around fivefold (Fig. 3d). Interestingly, expression of the EMT end stage marker Vimentin is not altered in the treated cells (Fig. 3b). As expected, pSMAD3 levels are upregulated in the treated cells. We conclude that treatment with TGFβ induces changes in PNT2-C2 cells that are generally characteristic of EMT.

PC3 cells have a mesenchymal morphology and they express very low levels of E-Cadherin, requiring long exposures to visualise the protein in western blot experiments. However, under these conditions western blotting clearly reveals that E-Cadherin protein levels are reduced following treatment with TGFβ (Fig. 3e). Moreover, the expression of Snail is increased in TGFβ-treated cells (Fig. 3e) as are the levels of pSMAD3 (Fig. 3f). However, as in the case of PNT2-C2 cells, Vimentin expression is not altered in the treated cells (Fig. 3e). In agreement with these results, quantitative PCR reveals that the CDH1 mRNA is downregulated in the treated cells (Fig. 3f), whereas the SNAI1 mRNA is upregulated (Fig. 3g). Similar results were obtained using another prostate cancer cell line (Fig. S2). Taken together, these data indicate that TGFβ induces changes in gene expression and cell behaviour in prostate cancer cells that are similar to those induced in PNT2-C2 cells.

### TGFβ increases PRH phosphorylation and decreases PRH protein levels

**Fig. 4:**
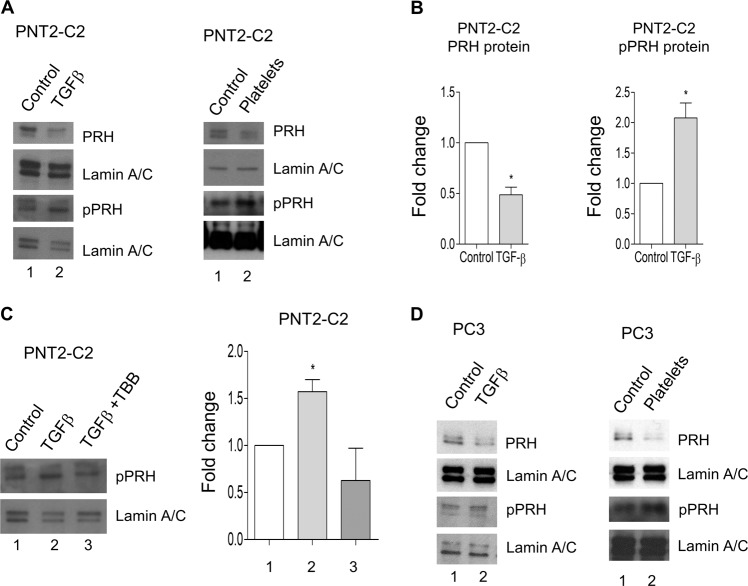
TGFβ increases PRH phosphorylation and downregulates PRH protein levels. **a** PNT2-C2 cells were treated with either 5 ng/ml TGFβ or platelets (1:1) or vehicle for 48 h. Western blotting was then used to examine PRH and pPRH levels using Lamin A/C as a loading control. **b** Three independent experiments performed as in **a** were quantified using densitometry. M + SD, *p < 0.01. **c** Left. PNT2-C2 cells were treated with 5 ng/ml TGFβ in the presence of absence of the Protein Kinase CK2 inhibitor TBB (10 μm) 48 h. Western blotting was then used to examine pPRH levels using Lamin A/C as a loading control. Right. Three independent experiments were quantified using densitometry. M + SD, *p < 0.01. **d** The experiment shown in part **a** was repeated using prostate cancer PC3 cells.

The experiments described above highlight the importance of TGFβ in EMT in these cells. Our previous work showed that increased PRH phosphorylation is associated with EMT-like changes in PNT2-C2 cells and that PRH depletion results in increased cell migration and invasion^26^. We therefore examined the effects of TGFβ and platelets on PRH phosphorylation and PRH protein levels. Western blotting reveals that treatment of PNT2-C2 cells with TGFβ or platelets results in a reduction in PRH protein levels (Fig. 4a, left and right panels, respectively). Quantification of multiple independent experiments shows that TGFβ treatment brings ~ 50% reduction in PRH protein levels in these cells (Fig. 4b, left). Moreover, treatment with TGFβ or platelets also bring about increases in PRH phosphorylation (Fig. 4a, lower panels). Quantification of multiple independent experiments shows that TGFβ treatment brings about a twofold increase in pPRH in these cells (Fig. 4b, right). These data suggest that TGFβ treatment results in the inactivation of PRH in these cells. To determine whether the increase in PRH phosphorylation is dependent on protein kinase CK2, we examined the effect of the CK2 inhibitor TBB on TGFβ-induced PRH phosphorylation. Figure 4c shows that in the absence of TBB treatment of PNT2-C2 cells with TGFβ increases pPRH levels. However, in the presence of TBB TGFβ treatment fails to increase pPRH levels (Fig. 4c). To determine whether TGFβ and platelets induce similar changes in PRH levels and PRH activity in prostate cancer cells, we next looked at the effects of these treatments on PC3 cells. Both treatments bring about reductions in PRH levels in PC3 cells and both treatments increase the levels of pPRH (Fig. 4d).

### TGFβ downregulates PRH mRNA levels and blocks the upregulation of E-cadherin expression by PRH

**Fig. 5:**
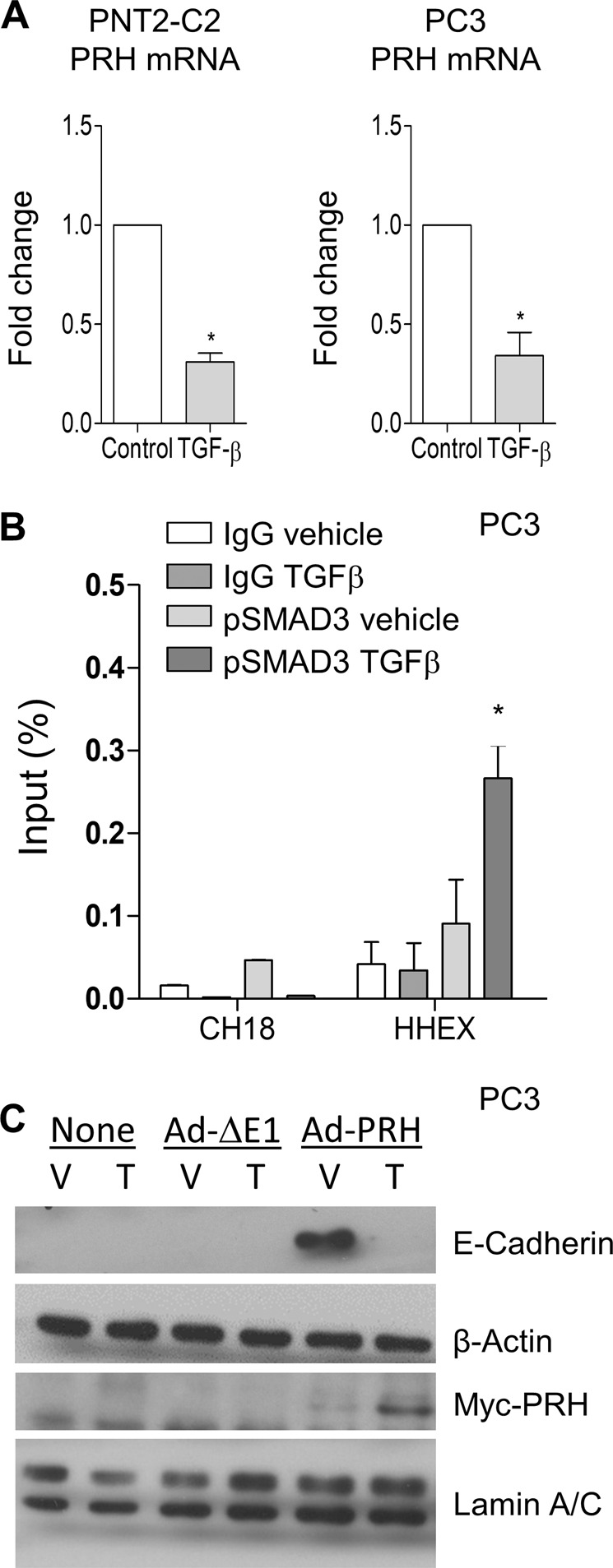
TGFβ downregulates PRH mRNA levels and prevents the activation of E-cadherin expression by PRH. **a** PNT2-C2 cells and PC3 cells were treated with 5 ng/ml TGFβ for 48 h. qRT-PCR was the used to determine PRH mRNA levels relative to GAPDH mRNA. The data shown are from three independent experiments each performed with triplicate PCR reactions. M + SD, *p < 0.01. **b** PC3 cells were infected with Ad-GFP or Ad-PRH at MOI 100 for 24 h then treated with 5 ng/ml TGFβ for a further 48 h. Chromatin immunoprecipitation was then performed using IgG or pSMAD3 antibodies followed by quantitative PCR using HHEX primers and Chromosome 18 gene desert primers as negative control. The results shown are from three independent experiments performed with triplicate PCR reactions. M + SD, *p < 0.01. **c** PC3 cells were infected with Ad-ΔE1 empty virus or Ad-PRH at MOI 100 for 24 h. The cells were then treated with vehicle (V), 5 ng/ml TGFβ (T), or control for 48 h. Western blotting was then performed for Myc-PRH and E-Cadherin with Lamin A/C and β-Actin as loading controls, respectively.

SMAD proteins have been reported to regulate expression of the PRH/HHEX gene in the mouse embryo^32^. To determine whether TGFβ regulates PRH gene expression, we treated PNT2-C2 cells with TGFβ and examined PRH mRNA levels using qRT-PCR. Figure 5a shows that treatment of these cells with TGFβ results in a significant reduction in PRH mRNA relative to glyceraldehyde-3-phosphate dehydrogenase (GAPDH) mRNA levels (Fig. 5a, left panel). Similarly, treatment of prostate cancer PC3 cells with TGFβ results in a decrease in PRH mRNA (Fig. 5a, right panel). To examine whether this change in gene expression correlates with changes in the binding of SMAD proteins at the PRH gene we made use of chromatin immunoprecipitation (ChIP) assays. PC3 cells were treated with TGFβ before fixation and ChIP using a pSMAD3 antibody or IgG as a negative control. Quantitative PCR was then used to determine whether PRH promoter sequences are preferentially pulled down. Following TGFβ treatment there is a statistically significant increase in enrichment in PRH promoter sequences (Fig. 5b) indicating that binding of pSMAD3 is increase under these conditions. In contrast, sequences from a gene desert region of chromosome 18 did not show any enrichment following TGFβ treatment (Fig. 5b).

As TGFβ treatment results in decreased PRH mRNA levels and decreased PRH protein levels, and since this correlates with increased binding of pSMAD3 to the PRH promoter, we next tested whether overexpression of PRH from a heterologous promoter could negate the effects of TGFβ on prostate cells. We overexpressed Myc-tagged PRH in PC3 cells under the control of the CMV promoter using a recombinant adenovirus (Ad-PRH) and then treated the cells with TGFβ. Consistent with our previous studies, overexpression of PRH resulted in increased expression of E-Cadherin (Fig. 5c). However, treatment of the PRH-overexpressing cells with TGFβ abrogated the effects of PRH on E-Cadherin protein levels (Fig. 5c, rightmost lane). These data show that the overexpression of PRH is insufficient to block the effects of TGFβ on these cells.

### PRH regulates multiple genes involved in EMT and TGFβ signalling

**Fig. 6:**
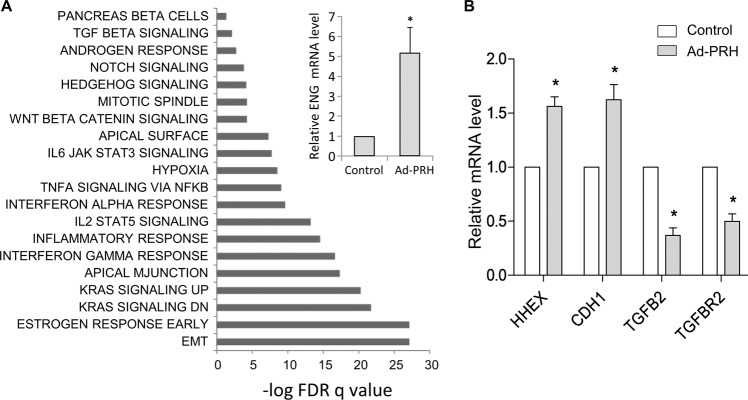
Differentially expressed gene sets in PNT2-C2 cells overexpressing PRH. **a** PNT2-C2 cells were infected with Ad-GFP or Ad-PRH at MOI 50 as in Fig. 5b. Changes in gene expression were then detected using microarrays and differentially expressed genes identified using SAM (statistical analysis of microarray, false discovery rate adjusted *p* < 0.01). Inset: relative expression of the TGFβ co-receptor Endoglin mRNA in the Ad-empty and Ad-PRH infected PNT2-C2 from cells from part A were determined by qRT-PCR and normalised to β-Actin mRNA (M + SD, *n* = 3, *p < 0.05). **b** PC3 cells were infected with Ad-empty and Ad-PRH as above and after 24 h mRNA levels were determined by qRT-PCR and normalised to GAPDH mRNA (M + SD, *n* = 3 independent experiments with triplicate PCR reactions, *p < 0.05).

Our previous work showed that PRH regulates the transcription of Endoglin, a TGFβ co-receptor that modulates TGFβ signalling^25^. Similarly, we have shown that PRH regulates the transcription of E-Cadherin^26^. This raises the question of whether PRH regulates other genes involved in TGFβ signalling and EMT. To investigate this we took an unbiased genome wide approach using microarrays. We overexpressed Myc-PRH in PNT2-C2 cells using a recombinant adenovirus as described above and used empty adenovirus as a control. We extracted mRNA from three independent experiments and three independent control infections and performed microarray experiments. In total, 6094 genes were significantly upregulated and 2373 genes downregulated between PRH-overexpressing cells and the control cells (fold change > 1.5). Gene ontology was then performed using gene set enrichment analysis (GSEA, for genes with false discovery rate (FDR) adjusted *p* < 0.1) using the Hallmark gene sets. Figure 6a shows significantly enriched gene sets. The gene set with the most significant alteration contains genes involved in EMT including VEGFC (x0.24), MMP3 (x0.45), and BMP1 (x1.7). Other significantly altered gene sets contain genes involved in oestrogen responses and the p53 pathway. Interestingly, TGFβ response genes also showed significant alteration including Endoglin (x1.6), SMAD6 (x1.8), and Noggin (x3.1). We validated these results using qRT-PCR to examine the expression of Endoglin mRNA (Fig. 6a, inset). To determine whether PRH overexpression would also alter the expression of TGFβ response genes in prostate cancer cells we infected PC3 cells with Ad-PRH or Ad-empty and we examined the expression of selected genes using qRT-PCR (Fig. 6b). As expected, PRH mRNA (HHEX) and CDH1 mRNA levels are increased following PRH overexpression. PRH mRNA levels are modestly increased at this multiplicity of infection (MOI). Interestingly, this modest overexpression results in a significant repression of TGFB2 and TGFBR2 mRNA levels. We conclude that PRH regulates the expression of multiple genes involved in EMT and TGFβ signalling in normal prostate cells and in prostate cancer cells.

## Discussion

Tumour cells are exposed to TGFβ from multiple sources both at their site of origin and during their metastasis to secondary sites. Prostate tumourigenesis is associated with inflammation, and immune cells produce a variety of signalling molecules that increase prostate cancer risk and aggressiveness including TGFβ^6^. Platelets are important in inflammation and in multiple disease states, and an elevated blood platelet count is indicative of a poor prognosis in breast cancer and other malignancies^30,33^. Several studies indicate that this is due in part at least to increased metastasis. For example, treatment of mouse colon carcinoma cells or mammary carcinoma cells with platelets prior to tail-vein injection increased the number of lung metastases and induced EMT-like changes in the treated cells^34^. The platelet-treated cells formed fewer metastases in mice lacking TGFβ in platelets and megakaryocytes than in wild-type mice, suggesting that platelet-derived TGFβ is important for the increased metastatic potential of these cells^34^. In addition, platelet-released ATP binds to P2Y2 receptors on blood vessel endothelial cells, inducing opening of the endothelial barrier and aiding, or allowing, the passage of cancer cell^27^. However, as well as activating P2Y2 receptors on endothelial cells, platelet-released nucleotides activate platelet P2Y12 receptors and this autocrine loop is known to enhance platelet aggregation and the release of TGFβ from platelet alpha granules^28^. TGFβ also induces opening of the endothelial barrier in this case by disrupting endothelial cell–cell junctions^35^.

The PRH protein regulates the proliferation of prostate epithelial cells and inhibits cell migration and invasion^25,26^. However, PRH phosphorylation inhibits PRH function and in BPH increased PRH phosphorylation correlates with increased cell proliferation^26^. In prostate cancer cells, PRH is also hyperphosphorylated^26^. Moreover, in these cells, PRH mRNA expression is reduced and in ~ 4% of prostate cancer patients the PRH gene is deleted. We have shown previously that experimental depletion of PRH in normal immortalised prostate epithelial cells induces EMT-like changes including increased cell migration and invasion and reduced expression of E-Cadherin^26^. In contrast, overexpression of PRH in prostate cancer cells inhibits proliferation and blocks cell migration and cell invasion^25^. We show that depletion of PRH in normal immortalised prostate epithelial cells increases platelet adhesion and increases the sensitivity of these cells to the stimulatory effects of platelets in an in vitro extravasation in which they must traverse a layer of endothelial cells and invade Matrigel. This suggests that the downregulation of PRH in BPH and in prostate cancer cells contributes to the acquisition of an invasive phenotype. The stimulatory effects of platelets on the extravasation of PRH-depleted cells is significantly reduced following treatment with an ATP diphosphohydrolase, indicating that ATP signalling is important in this process. The effects of platelets in this assay are also largely abrogated following the inhibition of TGFβ signalling. Moreover, TGFβ treatment increases the migratory potential of these cells and has similar effects on prostate cancer cells. These phenotypic changes are accompanied by the downregulation of E-Cadherin protein levels and the upregulation of Snail expression, changes that are indicative of the loss of epithelial characteristics and the gain of mesenchymal characteristics.

The mechanisms that enable TGFβ to induce EMT have been studied in some detail. However, here we show that TGFβ treatment increases PRH phosphorylation in normal immortalised prostate cells and prostate cancer cells. TGFβ treatment also downregulates PRH mRNA levels and PRH protein levels in these cells. Both of these mechanisms are likely to contribute to the induction of EMT and to be of relevance in prostate cancer progression. As PRH appears to function as a tumour suppressor in breast cancer cells and some other cancer cells^18^, it is likely that some or all of these mechanisms are important in the context of these diseases.

We have shown that PRH regulates the expression of multiple genes involved in EMT and TGFβ signalling in prostate cells. Thus, the downregulation of PRH activity by TGFβ has consequences for the expression of these genes and for the induction of EMT. We propose that the downregulation of PRH activity by TGFβ shapes the response of normal prostate cells and prostate cancer cells to immune signalling in the prostate and influences the response of cancer cells to these signals during metastasis. Moreover, changes in PRH activity will influence signalling from normal prostate cells and prostate cancer cells to immune cells. This feedback loop is likely to be important in cancer progression, and it represents a novel target for drug discovery and drug development. Drugs that prevent PRH phosphorylation such as the protein kinase inhibitor CX4945, for example, would be expected to downregulate some of the effects of TGFβ on these cells. Our future studies will address this question and the wider role of PRH-TGFβ co-regulation in other tumour types.

## Materials and methods

### Cell culture

PNT2-C2 cells were a kind gift from Professor Norman Maitland (University of York). PC3 cells and MCF-7 cells were obtained from ATCC. PNT2-C2 cells, PC3 cells, and MCF-7 cells were cultured in Roswell Park Memorial Institute (RPMI)-1640 medium (Sigma-Aldrich) supplemented with 10% fetal bovine serum (FBS), 2 mm l-glutamine, and 1% penicillin/streptomycin. HuVECs (PromoCell) were cultured in DMEM: F12 supplemented with 5 ng/ml epidermal growth factor, 10 ng/ml basic fibroblast growth factor, 20 mg/ml heparin, 1 mg/ml hydrocortisone, 250 ng/ml insulin, 1% penicillin/streptomycin, and 2% fetal calf serum. Cells were maintained in a humidified atmosphere at 37 °C and 5% CO_2_.

### PRH KD and overexpression

PRH expression was knocked down in PNT2-C2 cells by transfecting 5 × 10^6^ cells with 5 µg PRH49 and 5 µg PRH51 shRNA plasmids (Origene) using TransIT prostate transfection reagent (Mirus, Madison, WI, USA). Cells transfected with 10 µg SVC shRNA were used as a control. Cells were selected in media containing 1 µg/ml puromycin added 24 h post transfection and experiments were performed using cells harvested at day 10. PRH expression was knocked down in MCF-7 cells using an isopropyl b-d-1-thiogalactopyranoside-(IPTG) inducible PRH shRNA lentiviral construct (TRCN0000274008 Sigma-Aldrich) as described^25^. Cells transduced with the PRH shRNA virus or control scrambled shRNA virus were selected using puromycin and grown in the presence of 1 mm IPTG for 7 days to induce shRNA expression. PRH was overexpressed using an Adenoviral-Myc-PRH (Ad-PRH) construct^36^ at a MOI of 1: 100 for 24 h at 37 °C and 5% CO_2_. An adenovirus expressing β-galactosidase (Ad-β-gal) and empty virus were used as negative controls.

### Whole-cell extracts and western blotting

Western blotting was performed as described previously^37^ except that cells were lysed in lysis buffer (Cell Signalling) containing 1 µm phenylmethylsulfonyl fluoride and 1× PhoStop (Roche) with sonication on ice water for 10 minutes. Antibodies are listed in Table 1.

**Table 1:** Antibodies used in this study.

Antibody	Species	Source	Dilution
anti-Hex mAb, clone 4B7	Mouse	OriGene Technologies	1:1000
pPRH (YKN5) polyclonal	Rabbit	In house	1:2500
Phospho-Smad3 (Ser423/425) (C25A9) Rabbit mAb 9520	Rabbit	Cell Signalling Technology	1:1000
E-Cadherin (24E10) mAb 3195	Rabbit	Cell Signalling Technology	1:1000
β-Actin (D6A8) mAb 8457	Rabbit	Cell Signalling Technology	1:1000
Snail (C15D3) mAb 3879	Rabbit	Cell Signalling Technology	1:1000
Lamin A/C mAb 2032	Rabbit	Cell Signalling Technology	1:1000
Myc-Tag (9B11) mAb 2276	Mouse	Cell Signalling Technology	1:1000
Anti-mouse IgG, HRP conjugate AP160P	Rabbit	Sigma-Aldrich	1:5000
Anti-rabbit IgG, HRP conjugate 12–348	Goat	Sigma-Aldrich	1:5000

### Platelet preparation and adhesion assays

Platelets were prepared from freshly taken human blood as described previously^38^. Human blood was obtained from healthy drug-free volunteers, who gave full informed consent in accordance with the Declaration of Helsinki, and with approval from the local research ethics committee of the University of Bristol. For platelet adhesion assays, PNT2-C2 SVC control cell and PRH KD cells (1 × 10^5^ cells per well) were seeded in a tissue-culture treated 96-well plate (Corning), and incubated at 37 °C for 2 h. Platelets preloaded with 4 µm, Calcein-AM (Sigma-Aldrich) were then added and allowed to adhere to the cells for 45 min at 37 °C. Total fluorescence per well was measured using a microplate reader (Molecular Devices) at 485 nm. After three washes with phosphate-buffered saline (PBS), 100 µl of PBS was added to each well and the remaining fluorescence was read as above. Percentage platelet adhesion was calculated as: (remaining fluorescence−blank)/(total fluorescence−blank) × 100.

### Cell migration, invasion, and extravasation assays

Cell migration, cell invasion, and cell extravasation assays were performed as described previously^25^. In brief, chemotaxis assays were performed by seeding cells into Boyden chambers (Greiner Bio-One) in RPMI medium with 2% FBS in 24-well plates containing 800 µl RPMI with 10% FBS. The cells were fixed with 4% paraformaldehyde (Fisher) and stained with 2 µg/ml bisbenzimide (Sigma-Aldrich) before counting cells on the top and bottom of the filter using a Leica Q550 inverted epifluorescence microscope. Alternatively, migrated cells were incubated with RPMI containing 8 µm Calcein-AM for 45 minutes then removed using trypsin-ethylenediaminetetraacetic acid (EDTA) (37 °C) and placed in a black flat bottom 96-well plate and read using a fluorescent plate reader at an excitation wavelength of 485 nm and an emission wavelength of 520 nm. For invasion assays, 50% Matrigel (BD Biosciences) was added to each chamber and solidified at 37 °C for 1 h before seeding cells. Extravasation assays were performed after seeding HuVECs onto Matrigel and testing permeability to FITC-labelled dextran (Sigma-Aldrich). In some experiments cells were incubated with platelets in a 1:1 ratio before plating onto confluent HuVECs. SB431542 and apyrase were purchased from Sigma-Aldrich.

### Immunofluorescence microscopy

Cells were seeded onto 22 mm^2^ cover slips (Fisher) in six-well plates and left overnight to adhere. The cells were then fixed and permeabilised for immunofluorescent staining as described previously^39^. After mounting with VectaShield medium containing DAPI (Vector Labs), imaging was carried out using a Leica Q550 inverted epifluorescence microscope with DAPI, GFP, and TRITC filter sets.

### ChIP

ChIP was performed as described previously with minor modifications^25^. After fixation, cells were resuspended in 10 mm 4-(2-hydroxyethyl)-1-piperazineethanesulfonic acid (HEPES), pH 8.0; 10 mm EDTA; 0.5 mm EGTA; 0.25 % Triton X-100; 1 µm PFSM and 1 × PhoStop and incubated on rocker for 10 min at 4 °C. Following centrifugation for 5 min at 500 × *g*, 4 °C the pellet was resuspended in 10 mm HEPES, pH 8.0; 200 mm NaCl; 1 mm EDTA; 0.5 mm EGTA; 0.01 % Triton X-100; 1 µm PFSM and 1 × PhoStop and incubated at 4 °C for 10 minutes. After recentrifugation, the pellet was resuspended in 1 ml ChIP buffer (25 mm Tris-HCl, pH 8.0; 150 mm NaCl; 2 mm EDTA; 1 % Triton X-100; 0.25 % SDS; 1 µm PFSM and PhoStop) and disrupted using a Vibra cell sonicator with a micro tip. Chromatin–antibody complexes were precipitated using Dynabeads-Protein G (Invitrogen) at 4 °C for 4 h. The DNA was finally purified using AXYPREP magnetic PCR clean-up (Axygen Scientific).

### Quantitative PCR

Primers are listed in Table 2. mRNA extraction and RT-qPCR was performed as described previously^25,37^ in triplicate for the gene of interest with GAPDH as an internal control. Data was analysed using Rotorgene 6 software (Corbett Research; Rotorgene RG-3000) and fold change in expression determined using the efficiency adjusted quantitative PCR method^40^. For quantitative ChIP, PCR was performed in quadruplicate using 2× Rotor-Gene SYBR Green PCR Master Mix and the results expressed as percentage of input recovered.

**Table 2:** Primers used in this study.

Primer	Sequence
E-Cadherin F	GTAACGACGTTGCACCAACC
E-Cadherin R	AGCCAGCTTCTTGAAGCGAT
GAPDH F	TCCTTGGAGGCCATGTGGGCCAT
GAPDH R	TGATGACATCAAGAAGGTGGTGAAG
Snail F	GAGGCGGTGGCAGACTAG
Snail R	GACACATCGGTCAGACCA
PRH F	AAACCTCTACTCTGGAGCCC
PRH R	GGTCTGGTCGTTGGAGAATC
CH18 F	TTCAGTCTGGTGGTGGTGAACTA
CH18 R	GCCTTGGGAAATCCATCTTTT
−3472 HHEX F	TAGAGCAGCACAGGGTTTGA
−3472 HHEX R	GCCTTGATGTGGATGAGTGC
Smad7 F	GCAAATCCTTTCCATCTCCA
Smad7 R	TGCTTTGTGATTTGGCAGTC

### Microarrays

PNT2-C2 cells were infected with Ad-PRH or Ad-β-gal as a negative control as above. After 24 h mRNA was extracted using an RNeasy mini kit (Qiagen) and used for double-stranded cDNA synthesis (Roche). After labelling with Cy3 (NimbleGen One-Colour DNA labelling kit) the cDNA was hybridised to a Roche NimbleGen 12 × 135 K gene expression microarray. The array was then washed and scanned using the MS 200 Microarray Scanner. Data from three independent experiments were extracted and normalised by robust multi-array analysis using DEVA software (v1.2.1). Statistical analysis of microarray analysis using MeV software (v4.9) was used to identify genes showing significantly altered expression. Gene lists were generated using fold change < 1.5 and FDR adjusted *p* values of < 0.1. Gene ontology was assessed using GSEA^41,42^.

## Supplementary information

Supplementary material
