# Supplementary material for: Blood platelets stimulate cancer extravasation through TGFβ-mediated downregulation of PRH/HHEX
Source: Oncogenesis. 2020 Feb 4;9(2):10. doi: 10.1038/s41389-020-0189-0 (PMC7000753; doi:10.1038/s41389-020-0189-0)
Supplement: Supplementary file 1 — Supplementary material [file 41389_2020_189_MOESM1_ESM.pdf]

Figure S1

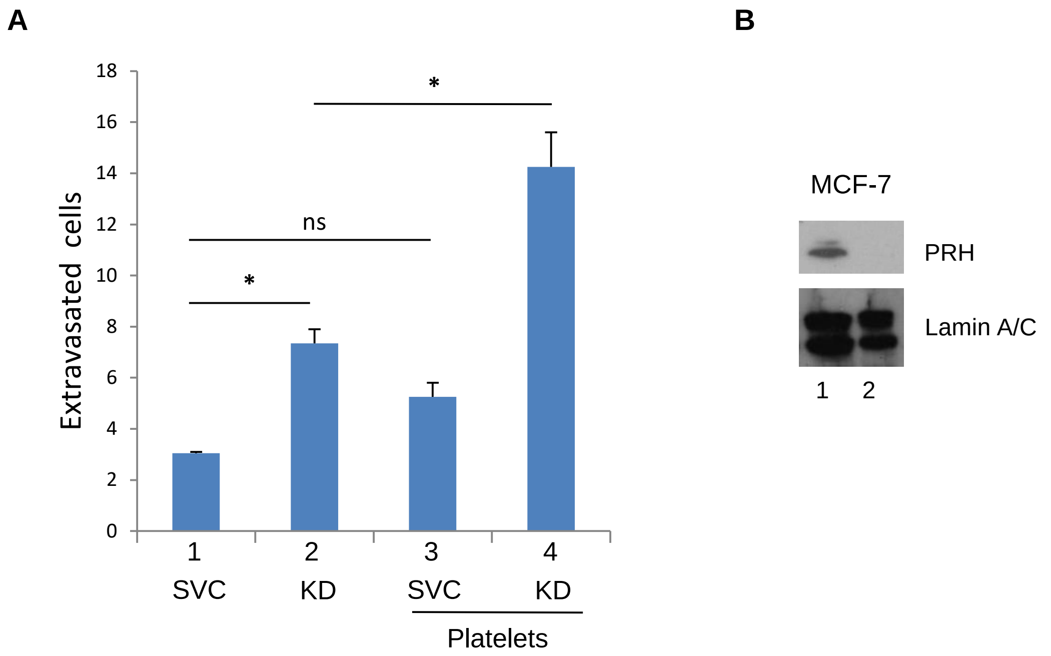

<u>Figure S1</u> <u>Blood platelets stimulate *in vitro* extravasation by MCF-7 PRH knockdown cells.</u> (A) Breast cancer MCF-7 cells expressing PRH shRNA (KD) or a scrambled vector control shRNA (SVC) were placed in a transwell chamber containing a confluent layer of HuVECs growing on Matrigel. Cells in lanes 3 and 4 were pre-incubated with blood platelets (1:1 cells:platelets) for 24 hrs prior to plating on HuVECs. After 48hrs the number of extravasated cells was determined by counting cells on the lower side of the transwell filter using microscopy. Cells per field, n=3 independent experiments, mean and standard deviation, ns=not significant, *p<0.01. (B) Western blot showing PRH protein levels in the cells from (A) with lamin A/C as loading control.

Figure S2

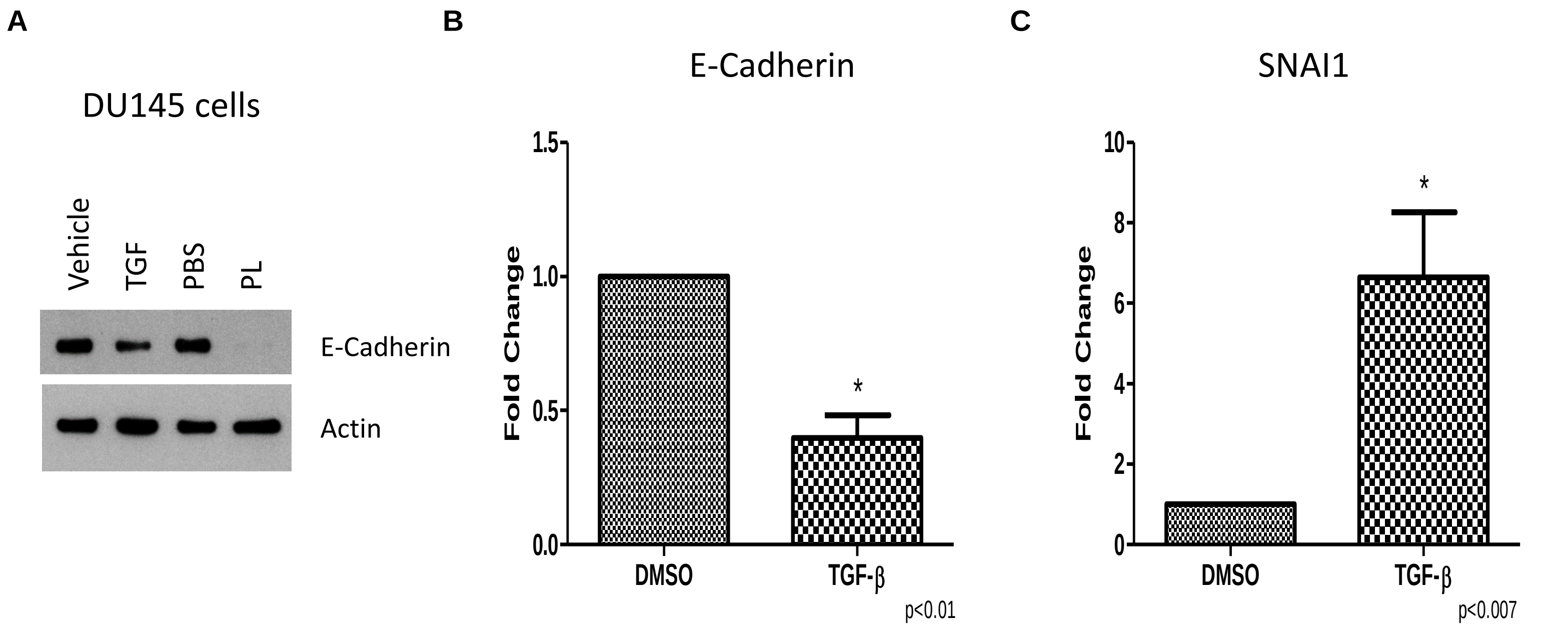

<u>Figure S2 TGFβ treatment down-regulates E-Cadherin expression and up-regulates SNAI1 mRNA levels in DU145 cells.</u> DU145 prostate cancer cells were treated with 5ng/ml TGFβ for 24 hrs (TGF) or 1:1 blood platelets (PL). E-Cadherin levels were then examined by Western blotting with β actin as a loading control (A) and following mRNA extraction qRT-PCR was used to determine CDH1 and SNAI1 mRNA levels (B and C, respectively). (B) and (C) show the results from three independent experiments with PCR performed in quadruplicate. Mean and standard deviation, *p<0.01.
